# Rehabilitation: Neurogenic Bone Loss after Spinal Cord Injury

**DOI:** 10.3390/biomedicines11092581

**Published:** 2023-09-20

**Authors:** Giovanna E. Leone, Donald C. Shields, Azizul Haque, Narendra L. Banik

**Affiliations:** 1 Department of Microbiology and Immunology, Medical University of South Carolina, Charleston, SC 29425, USA; gleone@email.sc.edu; 2 Department of Neurosurgery, Medical University of South Carolina, Charleston, SC 29425, USA; donshields@sbcglobal.net; 3 Ralph H. Johnson Veterans Administration Medical Center, Charleston, SC 29401, USA

**Keywords:** neurodegeneration, osteopenia, osteoporosis, sarcopenia, spinal cord injury

## Abstract

Osteoporosis is a common skeletal disorder which can severely limit one’s ability to complete daily tasks due to the increased risk of bone fractures, reducing quality of life. Spinal cord injury (SCI) can also result in osteoporosis and sarcopenia. Most individuals experience sarcopenia and osteoporosis due to advancing age; however, individuals with SCI experience more rapid and debilitating levels of muscle and bone loss due to neurogenic factors, musculoskeletal disuse, and cellular/molecular events. Thus, preserving and maintaining bone mass after SCI is crucial to decreasing the risk of fragility and fracture in vulnerable SCI populations. Recent studies have provided an improved understanding of the pathophysiology and risk factors related to musculoskeletal loss after SCI. Pharmacological and non-pharmacological therapies have also provided for the reduction in or elimination of neurogenic bone loss after SCI. This review article will discuss the pathophysiology and risk factors of muscle and bone loss after SCI, including the mechanisms that may lead to muscle and bone loss after SCI. This review will also focus on current and future pharmacological and non-pharmacological therapies for reducing or eliminating neurogenic bone loss following SCI.

## 1. Introduction

Spinal cord injury (SCI) is a severe neurological disorder that results from sudden and damaging impact to the spine and vertebrae [1,2]. SCI is one of the most commonly caused damages in vehicle injuries [3], but can also be caused by falls, athletic injuries, and various other reasons [4]. SCI impacts more than 10,000 individuals each year and poses a significant economic burden to the U.S [5]. SCI can be detrimental and life threatening, and while there are therapeutic modalities being studied, more research on how to mitigate the short- and long-term effects of SCI is still needed. The immediate impacts of SCI can vary and depend largely on the specific location and magnitude of the injury [1,6]. In general, the higher up the level of injury is to the spinal cord, the more severe the symptoms. Injuries to the spinal cord of any magnitude and location can have both localized and global effects on bone composition. The local effects include paralysis, reduced function in the lower body, and bone loss, most commonly in the femurs, tibias, fibulas, and pelvic bones. The global effects of SCI (i.e., neurogenic bone loss) include changes in neural signaling over time, which can lead to a disruption in bone remodeling throughout the body, not just in regions directly impacted by the SCI. The global effects of SCI may also include disruptions to bone vascularity, as there is a synergistic relationship between the skeletal and vascular systems. A decrease in bone vascularity and reduced neoangiogenesis can limit the healing capacity and progress of SCI rehabilitation modalities, and thus limit bone remodeling and repair [7]. People with a SCI are two to five times more likely to die prematurely than people without an SCI, and this carries substantial individual and societal costs. Short-term impacts often include gliosis, axonal damage, neuronal death, immobilization, and a loss of sensory and motor function, while long-term impacts include organ dysfunction, sarcopenia, osteopenia, bone fractures, and osteoporosis [1,4,8].

Demyelination and axonal degeneration are short-term but chronic outcomes of SCI, because they last for prolonged periods of time after the injury and are often irreversible [2,6]. Axonal degeneration occurs when the axons are lesioned, causing severe neuronal transmission deficits distal to the lesion site. This damage is furthered if the axon is lesioned in the central nervous system (CNS). Although there are potential therapeutic approaches to slowing axonal degeneration, this damage is usually permanent if the axonal lesion site is in the CNS [1,4]. Demyelination and a buildup of myelin debris are other immediate outcomes of SCI, which then lead to excessive levels of gliosis and glial scar formation [9,10]. These are just some of the immediate, short-term effects of SCI that come along with a multitude of long-term effects.

Many of the long-term outcomes of SCI are related to muscle and bone loss due to immobilization. Due to lack of physical activity and increased immobilization after one suffers from severe SCI, muscle and bone tissue severely decrease [11,12]. Osteoporosis is a common issue experienced after SCI and is defined as a skeletal disorder in which bone strength is compromised, leaving a person with a greater risk of fracture [13,14]. Individuals with osteoporosis experience large levels of osteopenia and are prone to fractures, which severely decrease quality of life and require substantial medical resources. Due to osteopenia after SCI, bone fractures are extremely common in individuals with SCI, because of their lower osteogenic load and increased bone demineralization [15,16,17]. The absolute causes of bone loss after SCI are not yet known; however, some of the possible causes are neurogenic factors, hormonal factors, and sarcopenia [15,18]. Immobility and disuse are other causes of osteopenia and sarcopenia in SCI patients due to the decrease in mechanical loading in the bone while one recovers from SCI. Sarcopenia, also known as muscle loss, has been linked to being a possible cause of osteopenia; however, more research is needed to evaluate the relationship between osteopenia and sarcopenia in SCI [11,19]. Diagnosis, prevention, and treatment for decreasing osteopenia and osteoporosis after SCI are critical to helping the thousands of individuals who suffer from SCI each year [15,17].

Therapies for reducing the negative outcomes of SCI are urgently needed. Although there has been promising research on therapies such as blocking 4-1BB and RANKL signaling [20,21], increasing Wnt signaling and calcium-regulated hormones [22,23], and loading of the bones and muscles [24], further research is still needed and there is research being conducted now on prospects for SCI treatments. The purpose of this review article is to discuss the pathophysiology of osteoporosis and determine the known treatments for bone loss and osteoporosis after SCI to reveal where more research needs to be conducted, as well as to cover the promising treatment options that are currently being studied.

## 2. Pathophysiology of Bone Loss after SCI

Individuals with complete paralysis after SCI show the most extensive bone loss and fracture risk [25,26]. Understanding the mechanisms that lead to bone loss and osteoporosis after SCI is important to determining how to slow bone loss after SCI. Common causes of bone loss after severe SCI are immobility and de-loading, which result in increased bone resorption and a decrease in osteoblast activity [4,27]. When one is immobile due to an injury, less stress is placed on the bones, leading to a direct response from other systems in the body, including the neurogenic and musculoskeletal systems [27]. Immobility has a direct effect on the musculoskeletal system, since it causes an increase in bone resorption and a decrease in osteoblast activity, resulting in osteopenia [4,27]. However, bone loss following SCI is believed to be distinct, as compared to the response to other disuse conditions in terms of both severity and mechanism. Although our focus is SCI, other factors secondary to SCI may also promote bone loss, including systemic hormonal changes, altered bone innervation, and impaired bone perfusion [26,28]. In an SCI study conducted on rats, significant bone loss was observed during a bone compartment analysis on the SCI animals compared to controls [11,29]. Overall, decreases in bone mineral content, trabecular structure, and bone mineral density were observed in all the SCI groups.

The next systems that immobilization and bone loss impact are the CNS, peripheral nervous system (PNS), and endocrine system. Bone cells have many nerve endings close to them, which greatly impact the CNS and PNS. Bone cells also connect the skeleton to the endocrine system through various receptors and neuromediators [27]. Skeletal loss may also promote sarcopenia and endocrine system dysfunction via multiple receptors and neuromediators, thus influencing the adipose tissue production of leptin and anorexigenics, which both affect bone remodeling [27,30]. Moreover, immobilization impacts skeletal vascularization, which is required for bone remodeling and osteoblast function. The resulting vasoconstriction further contributes to the muscular, endocrine, and nervous system impairments associated with osteoporosis in SCI patients.

The vascular system is a necessary contributor to osteogenesis after SCI. Neo-angiogenesis (i.e., the formation of new blood vessels) plays a crucial role in bone development after SCI, because it ensures that bone tissues are obtaining the necessary blood and oxygen supply to stimulate bone formation, maintenance, and repair [7]. Following SCI, individuals often experience disruptions to the circulatory system from mechanical trauma. Ischemia, hypoxia, and localized edema are potential secondary effects of SCI impacting the vascular system, thus impeding healing and rehabilitation [31]. The secondary effects of SCI on the vascular network not only potentially cause secondary injury and can further deteriorate bone and spinal cord tissue, but a reduced vascularity can also mitigate healing from SCI treatment [7,31]. Various SCI treatments, including cell transplantation, are ineffective if the local blood vessels are damaged, leading to a lack of oxygen and nutrients that the transplanted cells need for survival [31]. Pericytes and endothelial cells are important structures of the vascular system that play essential roles in angiogenesis; however, they cannot sustain and mediate angiogenesis to osteogenesis when there is damage to the blood vessels in the affected area [7,31]. Physical rehabilitation and therapeutic strategies, such as surgical anastomosis and exogenous pericyte cell transplantation, are available to help to stimulate angiogenesis after SCI [7]. Research is still limited on the effectiveness of therapy and rehabilitation for stimulating angiogenesis after SCI.

## 3. Disuse and Bone Loss after SCI

**Figure 1:**
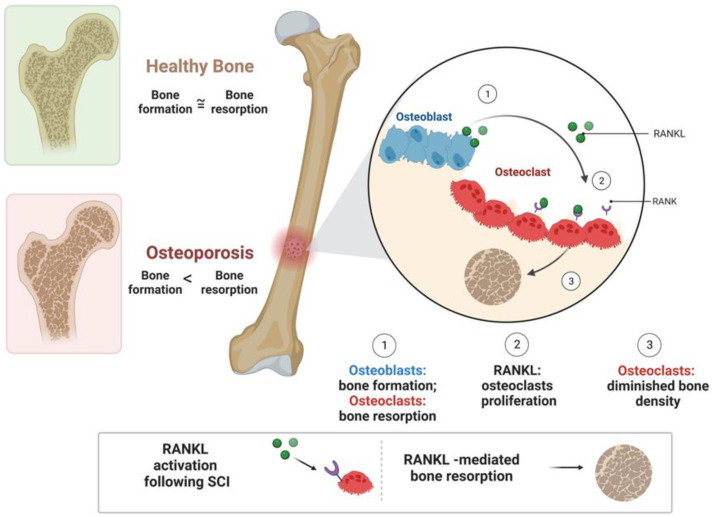
The pathophysiology of RANKL in bone resorption and osteoporosis after SCI. In healthy individuals, osteoblast/osteoclast activity provides for a healthy balance of bone formation and resorption. After SCI, RANKL increases osteoclastic activity, leading to increased bone resorption and osteoporosis. Figure created on Biorender.com.

The disuse of physical activity and loading is a main cause of osteopenia, which can cause localized bone loss and bone fractures, which are most commonly fractures of the distal femur and proximal tibia [32]. Bone loss after disuse is caused mainly by skeletal and mechanical unloading, meaning there is no pressure put on the skeleton, so it gradually and continually weakens [33]. The loss of bone appears to primarily be a consequence of decreased osteoblastic activity and number, although an increase in osteoclastic activity cannot be excluded (Figure 1). In studies performed on animals, de-loading has been found to be a direct cause of osteoblast activity and bone resorption [34]. After SCI, there are also multiple factors that can contribute to a decrease in mechanical loading on the skeletal tissue. Physical exertion stimulates osteoblast activity, which increases bone tissue via the mineralization of the skeleton. A lack of physical activity and skeletal loading (in many SCI patients due to paresis) is related to osteopenia and resulting fractures, most commonly of the distal femur and proximal tibia [32,33,34].

A lack of physical activity can also cause a decrease in the body mass (musculature and adipose tissues) load on the musculoskeletal system, thus creating less stimulation for osteoblast activity. Abdelrahman et al. examined the changes in total bone mineral content (BMC) and bone mineral density (BMD) in ten concentric sectors at the 4% site using tomography scans. They also analyzed the regional changes in BMC and cortical BMD in thirty-six polar sectors at the 66% site using linear mixed-effects models. They showed that the total BMC (*p* = 0.001) significantly decreased with time at the 4% site. Interestingly, the absolute losses of BMC and cortical BMD were similar at the 66% site. In a rat model, the SCI-induced bone changes observed were not solely attributable to bone loss [35], but also to suppress bone growth, suggesting that decreased whole-bone mechanical properties could be the result of changes in the spatial distribution of bone.

## 4. Risk Factors in SCI Individuals

The incidence and prevalence of SCI and its related complications have been increasing, with the incidence rate being estimated at from 15 to 40 cases per million worldwide [36,37,38]. The specific risk factors associated with SCI include age, gender, lifestyle, body mass index, and physical health conditions. SCIs are most common in males, who make up 78% of new SCI injuries in the U.S. [4,39]. Certain age ranges are more highly associated with SCI prevalence, including post-menopausal women and males aged 18–21 [32,40]. Post-menopausal women are likely at a higher risk of SCI due to the combination of having a higher risk for falls and a decreasing bone density. Males aged 18–21 commonly suffer from SCI due to lifestyles and behaviors that are common causes of SCI, such as contact sports and high falls [41,42]. Studies have suggested that the mean age of the SCI patient in developed countries is higher compared to that in developing countries over the same time period. Possible reasons for this are the aging of the populations in developed countries and/or the larger male-to-female ratio in developing countries in relation to developed countries [38,43]. Thus, it is likely that the elderly SCI populations in developed countries are suffering from additional complications such as bone fracture.

In recent years, epidemiological studies from countries worldwide have focused on traumatic SCIs, since the information about non-traumatic SCIs is limited and their risk factors are variable [41,42]. Traumatic incidents that are common risks of SCIs include sporting accidents, traffic accidents, and high falls [41,42]. Overall, there has been no obvious breakthrough in the determination of risk factors and clinical treatment of SCI and its associated complications; therefore, the emphasis has been on the prevention of traumatic SCIs.

Beyond structural loading, multiple factors, including an increased age, increased time since SCI, and lower body mass index, may be contributory risk factors to SCI [44,45]. Likewise, post-menopausal bone loss may exacerbate the skeletal effects following an SCI. Moreover, bone mineral density measurements shortly after an SCI are informative predictors of osteoporosis in the 12-month period following an SCI [32,40]. The type of SCI is also an important indicator of who will be at a greater risk of bone fractures. Recent findings have suggested that individuals who suffer from motor-complete SCIs have a higher risk of skeletal fractures; moreover, those who consume alcohol post-SCI are at a greater risk for fractures [46,47].

## 5. Cellular and Molecular Events following SCI

### 5.1. 4-1BB Signaling after Acute SCI

The receptor 4-1BB (also known as CD137) is a costimulatory and inflammatory receptor that is expressed on activated T cells [48] and some nonimmune cells, such as endothelial cells, glial cells, and neurons [49,50]. 4-1BB ligand (4-1BBL, also known as CD137L) is highly expressed on macrophages and antigen-presenting cells and can receive and transmit reverse signals into cells by binding to its receptor, 4-1BB [49,51,52]. The expressions of 4-1BB and 4-1BBL are upregulated on neuronal and immune cells following injury, and 4-1BB/4-1BBL signaling contributes to the progression of inflammation by controlling the communication of peripheral nerve fibers with cutaneous immune cells. Thus, 4-1BB/4-1BBL signaling might be involved in the regulation of glial and neuronal interaction, controlling neuroinflammation in the CNS. However, the underlying mechanisms and precise role of 4-1BB/4-1BBL signaling in the interplay of peripheral sensory neurons with immune cells are still not clear. Studies have shown the role of 4-1BB in the skeletal system in terms of osteoclast and function [53,54]. Increased bone resorption and decreased bone formation have also been found in aged mice compared to young mice. However, very little information is available on whether high-level 4-1BB/4-1BBL expression in bone marrow is associated with bone loss.

Increasing evidence has suggested that bone loss following an SCI may be affected by tumor necrosis factor receptor 4-1BB signaling. Animal studies have demonstrated that older mice have higher levels of 4-1BB in their bone marrow and have also been found to have a significantly greater bone loss than younger mice with less 4-1BB [21,55]. Targeted anti-4-1BB signaling may prevent bone loss in individuals who have just experienced an SCI. Likewise, anti-4-1BB-directed therapies are effective in treating various neoplasms; however, the treatment must be targeted directly to the tumor to limit the toxicity to bone marrow [56].

### 5.2. RANKL Signaling after SCI

Bone resorption and osteoclast function are also related to the release of the receptor activator of nuclear factor kappa-B ligand (RANKL) after SCI [57,58]. When individuals experience immobilization due to SCI, RANKL can cause much of the bone loss they experience [57]. The binding of RANKL to its receptor RANK can trigger osteoclast precursors to differentiate into osteoclasts (Figure 1). This process mainly depends on RANKL–RANK signaling, which is temporally regulated by various adaptor proteins and kinases. RANK is expressed in bone marrow mesenchymal stem cells (BMSCs) and is decreased during osteogenic differentiation [59]. RANKL expression can be reduced by the increased secretion of lipid-modified signaling glycoprotein, Wnt, which also stimulates osteoblast function and new bone cell production. Unfortunately, after SCI, Wnt is typically reduced, while RANKL is increased [57]. In addition to Wnt, ellagic acid (EA) has been found to block the interaction between RANK and RANKL, which inhibits the RANKL pathways and suppresses osteoclast activity [60].

### 5.3. Wnt Signaling after SCI

The Wnt/Beta-catenin pathway has been implicated in neuronal development and regeneration [61]. The central nervous system also utilizes this pathway after SCI for the regeneration of bone and CNS tissue via DNA replication, mitotic recombination, collagen/fibrin organization, and cell development [61,62,63]. Wnt-3a demonstrates a neuroprotective effect, contributing to neuropathic pain remission and neuronal survival. In animal studies, SCI subjects whose Wnt signals were blocked recovered three weeks after the animals without Wnt signal inhibition [62,64]. Moreover, SCI-related bone loss is reduced in rodents with increased Wnt signaling, related, in part, to reduced osteoclastogenesis and osteoclast activation. Furthermore, the Wnt pathway causes the secretion of glycoproteins from myofibers and satellite cells, with resulting increased levels of beta catenin, a multifunctional protein that promotes cell proliferation and muscle regeneration [63,65,66].

## 6. Calcium-Regulated Hormones in Bone Loss after SCI

Calcium and vitamin D play roles in bone health and regeneration. Immobilization, aging, and musculoskeletal disuse impede the metabolisms of vitamin D and calcium [33,67,68]. Although some controversy remains, there are reasonable data showing evidence that individuals who are either on a low calcium intake and/or have a vitamin D deficiency suffer from limited gastrointestinal calcium absorption, and may have an increased risk of fracture [68,69,70]. Individuals with SCI are also known to have a higher prevalence of vitamin D deficiency than the healthy population [70,71]. Studies have suggested that a significant depression in the ionized serum calcium concentration may trigger a secondary increase in the parathyroid hormone (PTH) concentration, which may result in an increased bone turnover in SCI individuals [72,73,74]. Vitamin D deficiency and abnormal PTH levels are also common in both acute and chronic SCI. The PTH levels are significantly reduced in SCI due to the hypercalcemia that accompanies increased bone resorption [70]. Thus, low PTH may contribute to SCI-induced bone loss. Insulin-like growth factor 1 can also play a role in blood calcium level regulation and changes in PTH in SCI [67,72]. The suppression of these hormonal factors, along with low estrogen/testosterone levels, are associated with bone and muscle atrophy [67]. Of note, PTH is not reduced significantly immediately after SCI, but instead slowly decreases over time. Thus, osteopenia secondary to SCI may play a decisive role in PTH reduction [67].

Individuals with SCI often show bone loss below the level of injury, and sometimes, it can happen throughout the body [75]. A recent study showed the progression of bone loss in SCI mice, which can begin as early as one week following injury in the hind limbs [72]. The total bone mineral density (BMD) and the BMD in areas above the level of injury are not significantly affected until the chronic stages of the injury. This study suggests that chronic SCI may induce a global dysregulation of bone homeostasis. Another study tested and compared the time course of bone loss following SCI in rats with different severities [76]. In severe SCI, rapid bone loss was observed as early as 2–3 weeks, and this bone loss was significant by 8 weeks. Thus, investigating how a loss of PTH following SCI affects the bones may help to develop effective therapies.

## 7. Bone Density and Fractures after SCI

Bone loss after SCI leads to an increased risk of low-impact fractures and significantly increases the morbidity and mortality of SCI individuals. Even though many severe SCI individuals employ wheelchairs for mobilization, they are still at risk for low-impact fractures [17,32,77]. Osteoporotic fractures are associated with chronic and disabling pain and can markedly increase the chances of death, especially in individuals over the age of 70 [78]. Common distal femur/proximal tibia fractures further limit mobility and impede rehabilitation [27,57]. Fractures after SCI are less common in the first year after injury, but as osteopenia and osteoporosis worsen over time, fractures become increasingly common [40,79]. Therefore, patients who experience SCI can benefit from bone density measurements and preventative treatments soon after injury to prevent future skeletal fracture. Adipocytes also secrete a protein, adiponectin, which may be a predictor of osteopenia in SCI patients. Adiponectin appears to induce osteoclast activity and osteoclastogenesis [80,81].

Studies evaluating SCI patients have found an inverse relationship between adiponectin levels and bone mineral density following SCI [34,80]. Adiponectin has also been identified as a marker for elevated fracture risks. A recent study characterized the time courses of cancellous and cortical bone deficits in a clinically relevant rodent SCI contusion model to determine the mechanisms of skeletal deterioration after SCI [82]. The findings from this study are very important from a clinical perspective, given that fracture incidence is associated with mortality in this population [83]. Overall, the authors found that severe cancellous bone loss occurred at the distal femur and proximal tibia within 2 weeks of SCI and thereafter temporally delayed cortical bone deficits similar to biphasic bone loss in human SCI.

Hormonal imbalance can also contribute to bone fracture and osteoporosis [84,85]. Estrogen plays a protective role in bone health. When estrogen levels decrease, such as after menopause, the risk of osteoporosis and bone loss rises. While post-menopausal women are more prone to osteoporosis and an increased risk of fracture, older men are not immune to a weakening of their bones due to hormonal changes. As men age, their bone density decreases, making fractures more likely [86,87]. In men, the aromatase enzyme converts testosterone into estrogen, and a loss of testosterone can impact this process and lead to bone density loss. Thus, the risk factors of age, duration of SCI, and neurological deficit negatively influence BMD, leading to fracture and bone loss.

## 8. Therapeutic Strategies for Neurogenic Bone Loss after SCI

### 8.1. Pharmacological Therapy

**Figure 2:**
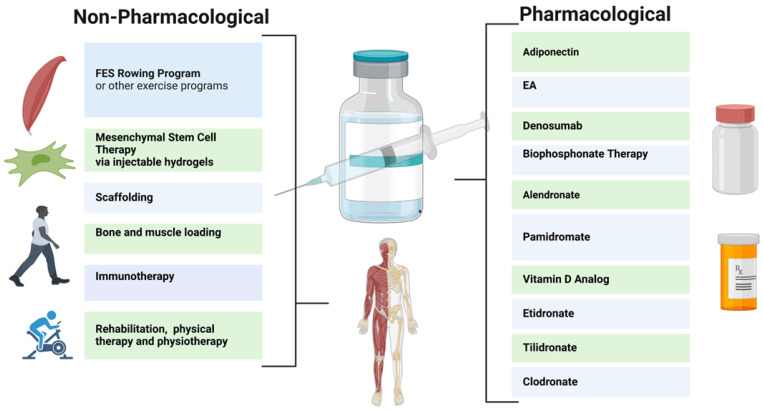
Rehabilitation Methods for Neurogenic Bone Loss After SCI. Figure created on https://www.biorender.com/.

Pharmacological therapies for the bone loss in SCI individuals have been relatively ineffective. While vitamin D supplementation is commonly used to restore the vitamin D levels in SCI individuals with a vitamin D deficiency, it has not been effective in preventing and restoring bone loss [88]. Thus, multiple pharmacological strategies may provide benefits for neurogenic bone loss after SCI. For example, ellagic acid (EA) has been found to bind to RANKL and downregulate osteoclast activity, although this endogenous compound may produce negative side effects at elevated concentrations [60,89,90]. Bisphosphonates and Denosumab have also been evaluated for their prevention of the loss of bone mass after SCI (Figure 2). Bisphosphonates act to slow bone loss by inhibiting bone resorption; these include Etidronate, Clodronate, Pamidronate, Tiludronate, and Alendronate [44,91,92]. Bisphosphonates used in SCI patients have been shown to reduce the risk of hip fractures (but not knee fractures) [29,93].

Despite some success, the effects of bisphosphonates have been inconsistent. Clodronate, Etidronate, and Tiludronate have been shown to yield increased bone mass in less than one year post injury (Figure 2), whereas Alendronate improved bone mass in more than one year after injury [44]. However, Pamidronate was not shown to improve bone mass in this study. In addition, the prolonged use of bisphosphonate therapy may produce adverse effects such as osteonecrosis of the jaw; thus, judicial administration is advised [94]. These therapies are currently available in oral or intravenous administrations, and single annualendroal bisphosphonate injections may be available for SCI patients in the future [29,94,95]. In a recent larger clinical trial on patients with chronic SCI, Teriparatide treatment was used, which resulted in a significant increase in spine BMD at 1 year and further improvements in the hip at 2 years [96,97]. Furthermore, Denosumab, a monoclonal antibody to RANKL, is FDA approved for osteoporosis treatment [98,99]. Denosumab prevents bone loss in SCI patients via the inhibition of osteoclast activity via the RANKL pathway, however, it must be frequently administered [93,100,101,102]. Denosumab thus reduces bone resorption and increases bone mineral density, reducing the risk of fractures.

### 8.2. Nonpharmacological Therapy 

Pharmacological therapies to date are limited, as they do not provide a significant restoration of damaged spinal cord parenchyma. Therefore, non-pharmacological approaches, such as mesenchymal stem cell (MSC) therapy, physiotherapy, immunotherapy, injectable hydrogels, and stem cell secretome therapy, are under consideration [103,104]. MSCs from the bone marrow, umbilical cord, and/or adipose tissue may reduce inflammation and provide neuroprotective effects to prevent further injury to the spinal cord near the impact site. Injectable hydrogels, which facilitate MSC targeting, are also being studied [105].

These therapies, in conjunction with weight-bearing rehabilitation, may be increasingly employed to decrease osteopenia in patients with SCI [106,107]. Following SCI, a primary catalyst behind bone loss is the decrease in mechanical loading. When individuals with SCI cease weight-bearing activities, they face a heightened susceptibility to rapid bone resorption and osteocyte apoptosis, frequently leading to the development of osteoporosis. Engaging in any form of mechanical loading on the skeletal system, including compression, tension, torsion, or bending, will uphold bone density and promote bone mass recovery [108]. Therapies aimed at this axial loading encompass activities such as walking, jogging, and jumping. Rehabilitations that stimulate mechanical loading are practical, non-invasive, and economical methods for stimulating bone regeneration [109]. Rehabilitation improves mechanical loading by exposing tissues to a range of strains and forces, prompting osteocytes to sense stress and begin to stimulate regeneration [110]. Reciprocally, the subjection of mechanical loading on tissues from rehabilitation has been shown to be an effective therapy for tissue regeneration, which ultimately improves the bone’s capacity for mechanical loading [109]. Rehabilitation also enhances mechanical loading by modifying and improving vascularization, thereby facilitating bone growth. Therapies with an increased musculoskeletal load have proven effective; however, this approach is limited in patients who are wheelchair-bound after SCI. Stand-up wheelchairs, standing frames, and suspended treadmills can provide useful alternatives [11,111,112]. Physical activity, which inherently stimulates the axial loading of the tibia, femur, and axial skeleton, may also promote bone density after SCI by improving bone vascularization and osteoblast activity [4,112].

Static loading and prone position muscle stimulation appear to be less effective techniques for the attenuation of bone loss after SCI [4,113]. Thus, functional electrical stimulation (FES) rowing following SCI has been evaluated. FES rowing employs cyclical exercise patterns coupled with electrical stimulation to simulate the functional motor patterns otherwise impaired by SCI. Rowing allows for paraparetic SCI patients to exercise in a sitting position (in some cases with a cycle ergometer), coordinating their upper body movements with the electrical stimulation of the lower body muscle groups to recreate the effects of full-body exercise [44,113,114]. In one trial, the bone loss in the distal femur and tibia appeared to be reduced in the majority of participants after 30 sessions; however, other results have suggested that bone loss is ameliorated with muscle electrical stimulation alone. Non-mechanical load-bearing exercises such as swimming and cycling are weaker therapies in terms of reducing bone loss; however, they have still been shown to be effective at maintaining muscle mass, which can indirectly reduce fracture risk. Further studies are therefore needed to determine how these therapies can be best implemented for SCI individuals who are wheelchair-bound. It is important to note that the extent of improvement in mechanical loading after SCI is highly dependent on individual aspects such as the severity of the injury. To yield the best results, rehabilitation should be started early, be consistent, and be tailored to individual needs and goals.

## 9. Conclusions

While SCI can lead to an irreversible loss of motor control and sensations below the level of trauma, the secondary consequences and complications associated with chronic SCI may be subjected to a devised repair strategy. SCI individuals experience a significant number of complications, including muscle wasting, osteopenia or osteoporosis, hormone dysregulation, cardiovascular problems, reduced angiogenesis, and immune deficiency. Although many of these complications appear soon after the injury, very little is known about the exact mechanism(s) underlying their development and progression overtime.

In general, SCI severely limits one’s physical and functional capacity due to the many limitations caused after an injury. A significant comorbidity related to SCI is neurogenic bone loss, which predisposes these individuals to osteoporosis and fractures. To reduce the risks of long bone fractures after SCI, pharmacological approaches, including the administration of ellagic acid, Adiponectin, Denosumab, and bisphosphonates, are being evaluated. Non-pharmacological treatments further augment bone density; these include exercise therapies such as FES rowing, bone loading, physiotherapy, and mesenchymal stem cell therapy. The application of both types of therapeutic approaches must be appropriately tailored for individual SCI patients in relation to the time after injury, side-effects, and other patient-specific comorbidities. In addition, studies are needed to develop novel combination approaches and determine the most effective therapies and prevention methods for osteoporosis in people with SCI.

## Data Availability

The data used to support the findings of this manuscript are available from the corresponding authors upon reasonable written request after the publication.
